# Establishment of prognostic model of bladder cancer based on apoptosis-related genes, in which *P4HB* promotes BLCA progression

**DOI:** 10.1186/s12894-023-01331-5

**Published:** 2023-10-16

**Authors:** Zhenhai Zou, Zhong Li, Wei Sun, Wuyue Gao, Beibei Liu, Jianmin Liu, Yuanyuan Guo

**Affiliations:** Department of Urology, the First Affiliated Hospital of Bengbu Medical College, No.287, Changhuai Road, Longzihu, Bengbu, 233040 Anhui China

**Keywords:** Apoptosis-related genes, Bladder carcinoma, Prognostic model, Immune infiltration, *P4HB*

## Abstract

**Background:**

A variety of apoptosis genes have been confirmed to be related to the occurrence and development of bladder cancer patients, but few studies have paid attention to their significance in the prognosis of bladder cancer. Therefore, this study explored the value of apoptosis-related genes in the prognosis of BLCA by using the data in TCGA database.

**Methods:**

We downloaded the mRNA expression profiles and corresponding clinical data of bladder cancer patients from TCGA database, and obtained 2411 apoptosis-related genes from Deathbase database. Screening out differentially expressed apoptosis-related genes. Cox regression was used to determine the prognostic value of apoptosis-related genes, and then a prognostic risk model was developed. A nomogram based on risk model was constructed to predict the prognosis of bladder cancer patients. At the same time, immune infiltration correlation analysis of genes in the prognosis model.

**Results:**

A prognostic model composed of 12 apoptosis-related genes was constructed. According to the risk score calculated by the model, patients were divided into high-risk group and low-risk group. There are significant differences in the expression of immune cells, immune function and immune checkpoint molecules between high-risk group and low-risk group. *P4HB* may promote bladder cancer progression.

**Conclusion:**

Based on the differential expression of apoptosis-related genes, we established a risk model to predict the prognosis of bladder cancer patients, in which *P4HB* promotes BLCA progression.

**Supplementary Information:**

The online version contains supplementary material available at 10.1186/s12894-023-01331-5.

## Introduction

Bladder cancer (BLCA) is the most common malignant tumor of the urinary system, the morbidity and mortality have increased in recent years [1]. Approximately 70% of bladder cancers are non-muscle invasive bladder cancer, and the main treatment is transurethral bladder tumor resection (TURBT) combined with intravesical chemotherapy [2]. Unfortunately, there are still 50% to 70% of NMIBC patients will relapse within 5 years after first-line therapy, and around 10% to 30% of patients exhibit progress to MIBC [3]. Although the diagnosis and treatment technology of bladder cancer has been improved, the five-year survival rate of BLCA patients is still only 70% due to its high recurrence and metastasis rate [4]. At present, there is still no clinical indicator that can accurately predict the prognosis of BLCA patients, though Next Generation Sequencing (NGS) has been used widely for the diagnosis of diseases. So, it is crucial to identify new prognostic markers and develop a prognosis predicting model of BLCA based on these gene markers.

TCGA (The cancer genome atlas) is composed of National Cancer Institute (NCI) and National Human Genome Research Institute (NHGRI, In 2006, the National Human Genome Institute of the United States jointly launched the project, which collected clinical data, genome variation, mRNA expression, miRNA expression, methylation and other data of various human cancers (including subtypes of tumors) [5], providing a basis for using bioinformatics analysis to predict related Apoptosis, a highly-regulated form of programmed cell death, plays a key role in the development and homeostasis of multicellular organisms [6]. Defects in apoptosis are associated with a variety of diseases, including autoimmune diseases, neurodegenerative diseases, bacterial and viral diseases, heart disease and cancer [7]. Two common initiation approach of apoptosis are the intrinsic (mitochondrial) pathway and the extrinsic (death receptor) pathway, which ultimately lead to cause apoptosis together [8]. It is achieved through the mechanism of programmed cell death, through a complex series of molecular signalling and biochemical reactions that culminate in nuclear chromosome breaks, lysis of cell membranes, and ultimately phagocytosis and degradation [9]. Apoptosis occurs through a complex series of molecular signalling and biochemical reactions, culminating in nuclear chromosome breaks, cell membrane lysis, and ultimately phagocytosis and degradation [10]. The microenvironment plays an important role in the regulation of apoptosis in cancer cells, such as the vasculature, stroma, oxygen concentration, pH and cell surface molecules in the tumour microenvironment. These factors can directly or indirectly inhibit apoptosis of tumour cells, which is conducive to tumour growth, proliferation and metastasis [11]. However, the relationship between apoptosis-related genes and bladder cancer prognosis has not been reported, and little is known about its association with the immune microenvironment of BLCA.

Therefore, in this study, the expression levels of apoptotic genes in bladder cancer tissues and their roles in predicting the prognosis of bladder cancer were identified and the risk prognosis model of bladder cancer related to apoptotic genes was constructed by using the data of bladder cancer patients in TCGA database,,aiming to provide a theoretical basis for the study of molecular mechanisms and the exploration of diagnostic and prognostic markers for bladder cancer.

## Materials and methods

### Data collection

The mRNA expression data and clinical information of BLCA patients were obtained from the TCGA database (https://portal.gdc.cancer.gov//) and GSE13507 profile downloaded from the GEO database (https://www.ncbi.nlm.nih.gov/geo/). Human apoptosis-related genes were downloaded from GSEA (https://www.gsea-msigdb.org/gsea/index.jsp) and these gene sets are displayed in the Supplementary Table S1.

### Differentially expressed ARGs and the enrichment analysis

The limma package and the Wilcoxon signed-rank test in R software 3.6.2 (| log 2 FC |> 1, FDR < 0.05) were used to obtain significantly differentially expressed apoptosis-related genes in bladder cancer patients. Volcano maps and heat maps were drew by the pheatmap and ggpubr packages. Gene ontology (GO) enrichment and Kyoto Encyclopedia of Genes and Genomes (KEGG) pathway analysis were performed to assess the potential biological function of differentially expressed apoptosis-related genes, ggplot2, DOSE, clusterProfiler, enrichplot, GOplot and other R software packages were used to visualize them in order to explore the potential molecular mechanisms of differentially expressed ARGs.

### Establishment of prognostic risk model

Identified ARGs related to prognosis by univariate Cox regression analysis. GSEA analysis further evaluated their related pathways and molecular mechanisms in bladder cancer, and a box plot was constructed to show the differential expression of ARGs related to prognosis in tumor tissues and normal tissues. Multivariate Cox regression analysis was then used to establish the risk prognostic model and obtain the corresponding regression coefficients. The regression coefficient was used to calculate the corresponding risk score, and the formula for calculating the risk score (RiskScore) is: RiskScore = gene expression 1 × Coef1 + gene expression 2 × Coef2 + .. + gene expression amount n × Coefn (Coef: regression coefficient in multivariate Cox regression analysis, n: total number of apoptosis-related genes associated with prognosis). Patients were divided into high-risk and low-risk groups based on the calculated median risk score. Prognostic models were constructed using the "glmnet" package in R.

### Validation of the prognostic risk model

Utilizing Kaplan–Meier (KM) curve and the area under the receiver operating characteristic (ROC) curve (AUC) to assess the predictive value of the prognostic model. Univariate and multivariate prognostic analysis, R package timeROC, were used to determine whether the risk score could be an independent indicator of OS in the BLCA patients. Finally, a nomogram based risk model was used to predict the prognosis of patients with bladder cancer.

### External validation of the model

Risk scores were calculated based on the expression of ARGs in the GEO database and clinical specimen data, categorised into high-risk and low-risk groups based on the scores. Kaplan–Meier (KM) and area under the curve (AUC) of subjects' work characteristics (ROC) to assess the predictive value of the prognostic model.

### Immune invasion level analysis

Based on TIMER database (https://cistrome.shinyapps.io/timer) to observe the expression abundance of immune cells between high-risk group and low-risk group, GSVA and GSEABase were used to analyze immune cells and immune function. Finally, the difference analysis of immune checkpoint was carried out to observe the significance of the model in immunotherapy.

### Clinical specimen

Bladder cancer tissue and paracancerous tissue were obtained from 86 bladder cancer patients in the First Affiliated Hospital of Bengbu Medical College from October 2017 to December 2020. None of them received preoperative radiotherapy or preoperative chemotherapy. All specimens were processed according to ethical and legal standards. This study was approved by the Ethics Committee of the First Affiliated Hospital of Bengbu Medical College [2022] No. 144.

### Cell line and culture

Bladder cancer cell line T24 and 5637 were From Shanghai Cell Bank of China Academy of Sciences cultured in RPMI1640 medium containing 10% fetal bovine serum. The cells were digested and passaged with 0.25% trypsin when the cell fusion was around 80%, with fluid changes or passages every 2 days.

### Reverse transcription-quantitative polymerase chain reaction (RT-qPCR)

Total RNA was isolated from tissues and cells using Trizol reagent and reverse transcribed into complementary deoxyribonucleic acid (cDNA). After pre-denaturation at 95 °C for 1 min, denaturation at 95 °C for 20 s and annealing at 60 °C for 20 s, a total of 40 cycles, the 2ΔΔCt method was used to calculate the relative expression of the target genes. Primers were designed using Primer 5. 0 (https://www.premierbiosoft.com/primerdesign/).

### Cell counting kit-8 assay

Cells were inoculated in 96-well plates and cultured for 24 h, 48 h and 72 h, respectively. 10μL CCK8 solution was added to each well, protected from light and cultured for 1 h at 37℃. The absorbance value of the cells at 450 nm (OD450nm value) was detected by enzyme marker and the experiment was repeated 3 times.

### Transwell assay

In the cell invasion assay, preparing Matrigel gel on the upper chamber surface, the cells were adjusted to 2 × 10^4cells/ml with serum-free medium and 100 μL was aspirated and added to the upper chamber. In the lower chamber, 1640 medium containing 20% fetal bovine serum was added. 24 h of incubation at 37 °C, the cells and Matrigel gel were gently removed from the upper chamber, fixed in 4% paraformaldehyde for 30 min and then stained with crystalline violet, photographed and analysed under a microscope. For the cell migration assay, no Matrigel gel was added to the upper chamber of the Transwell and the rest of the steps were the same as for the cell invasion assay, which was repeated three times.

### Flow cytometry

Collect the transfected cells, take 50,000–100,000 resuspended cells, centrifuge at 1000 g for 5 min and discard the supernatant. 195μLAnnexin V-FITC conjugate was added to resuspend the cells. Add 5μL Annexin V-FITC, 10μL of propidium iodide staining solution, mix well and inc ubate for 10-20 min at room temperature, flow-on assay, and repeat the experiment 3 times.

### Statistical methods

Gene expression data were normalized by log2 transformation. R software 3.6.2 and Perl language package were used to construct graphs, and Wilcoxon test was used to analyze differentially expressed genes in paracancerous tissues and tumor tissues. *P* ≤ 0.05 was considered statistically significant.

## Results

### Differentially expressed apoptosis-related genes and enrichment analysis

**Fig. 1:**
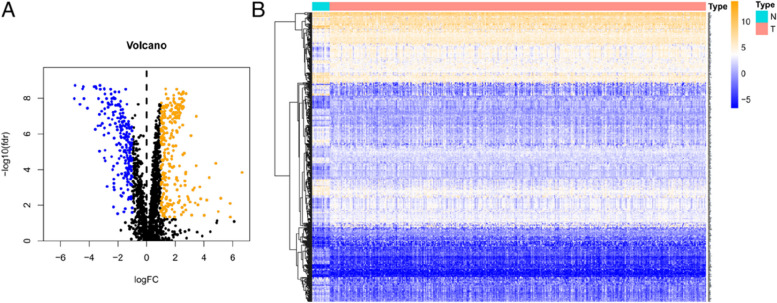
Differentially expressed ARGs. **A** Volcano plot of differentially expressed ARGs. Yellow represents high expression, blue represents low expression, black represents no difference between BLCA and normal tissues. **B** The heatmap of 481 differentially expressed ARGs

**Fig. 2:**
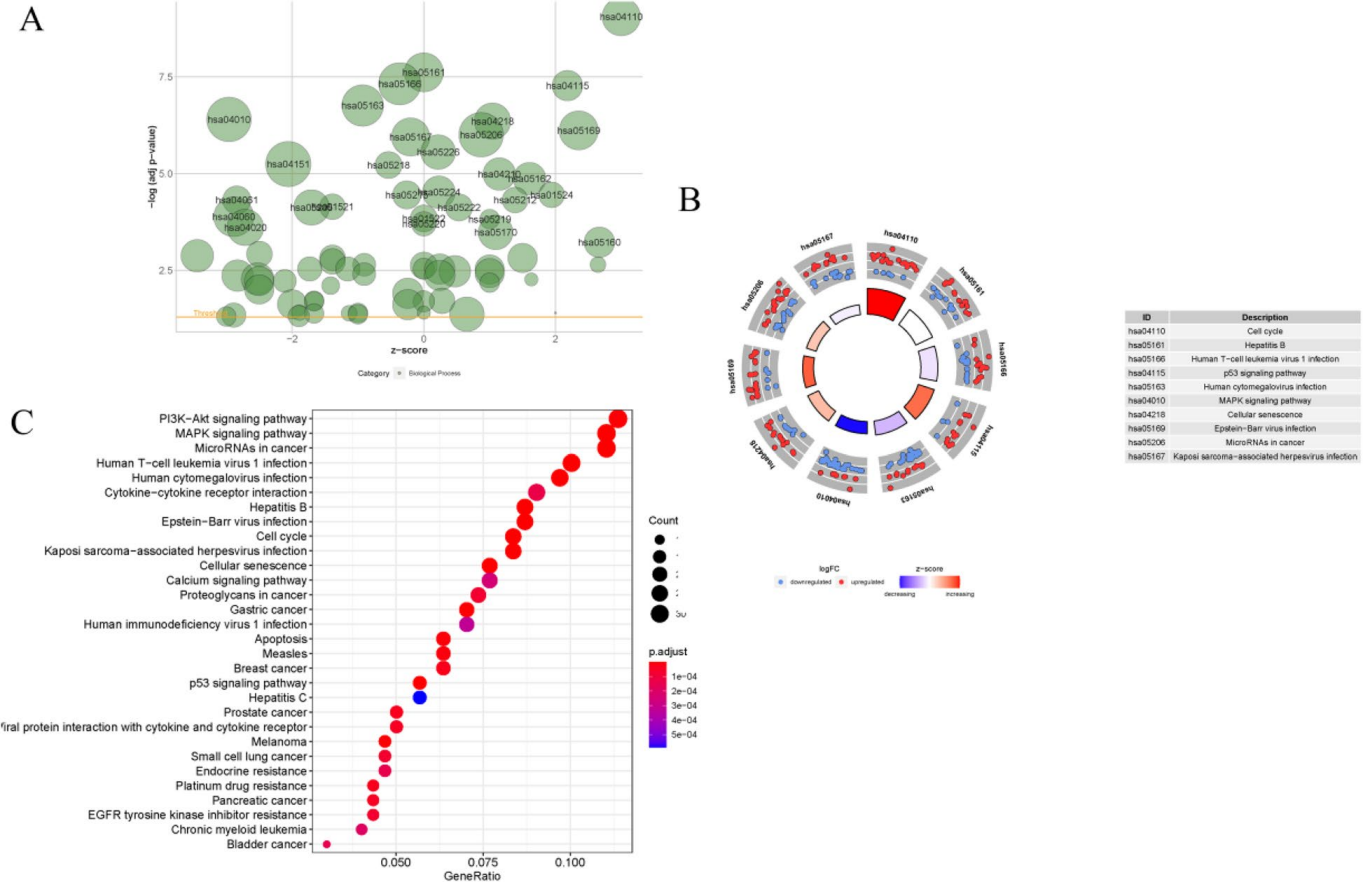
GO and KEGG enrichment analyses. **A** The bubble polt of GO analyses **B** the circle plot of GO analyses. **C** Results from KEGG analysis are presented in the bubble plot (from www.kegg.jp/kegg/kegg1.html)

mRNA data and corresponding clinicopathological features of 414 bladder cancer (BLCA) and 19 normal controls were downloaded from TCGA database. 2411 apoptosis-related genes were obtained from Deathbase database. With *FDR* < *0.05* and | log2 (Fold Change) |> 1 as thresholds, a total of 481 differentially expressed apoptosis-related genes were identified between tumor tissue and nontumor tissues, including 259 up-regulated genes and 222 down-regulated genes. The volcano map and heat map of the differentially expressed genes are shown in Fig. 1A and B. The differentially expressed apoptosis-related genes were analyzed for GO function and KEGG pathway. The results of GO functional enrichment showed that these genes were closely related to Cell cycle, Hepatitis B, Human T-cell leukemia virus 1 infection, p53 signaling pathway, Human cytomegalovirus infection, MAPK signaling pathway, Cellular senescence, etc. (Fig. 2A and B). KEGG pathway analysis showed that these genes were mainly enriched in oncogenic pathways such as PI3K-AKT and MAPK, and were associated with miRNAs and cells (Fig. 2C).

### Construction of prognostic risk model

**Fig. 3:**
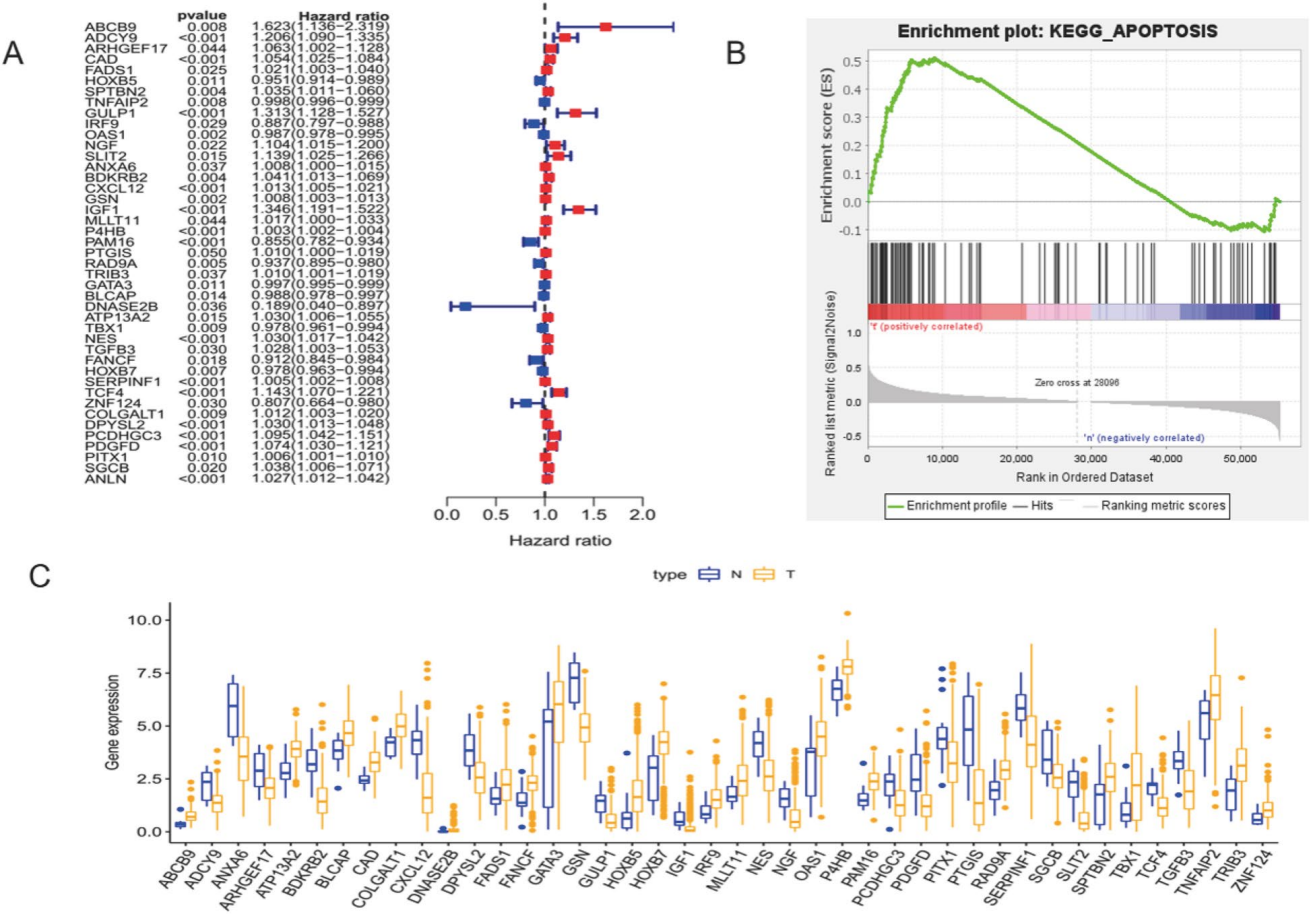
Univariate Cox,GSEA enrichment and boxplot. **A** Forest plot showing the results of the univariate Cox regression analysis **B** GSEA enrichment plot of KEGG pathways. **C** The boxplot of 44 identified ARGs. Yellow represents BLCA tissues, while blue represents normal tissues

The 481 differentially expressed genes were analyzed by univariate COX analysis, and 44 apoptosis-related genes significantly related to prognosis were obtained *(P* < *0.05)*, the results were plotted in the corresponding forest diagram (Fig. 3A). GSEA analysis showed that these genes were enriched in apoptosis-related pathways, and the corresponding boxplots were plotted according to gene expression (Fig. 3B and C).

**Fig. 4:**
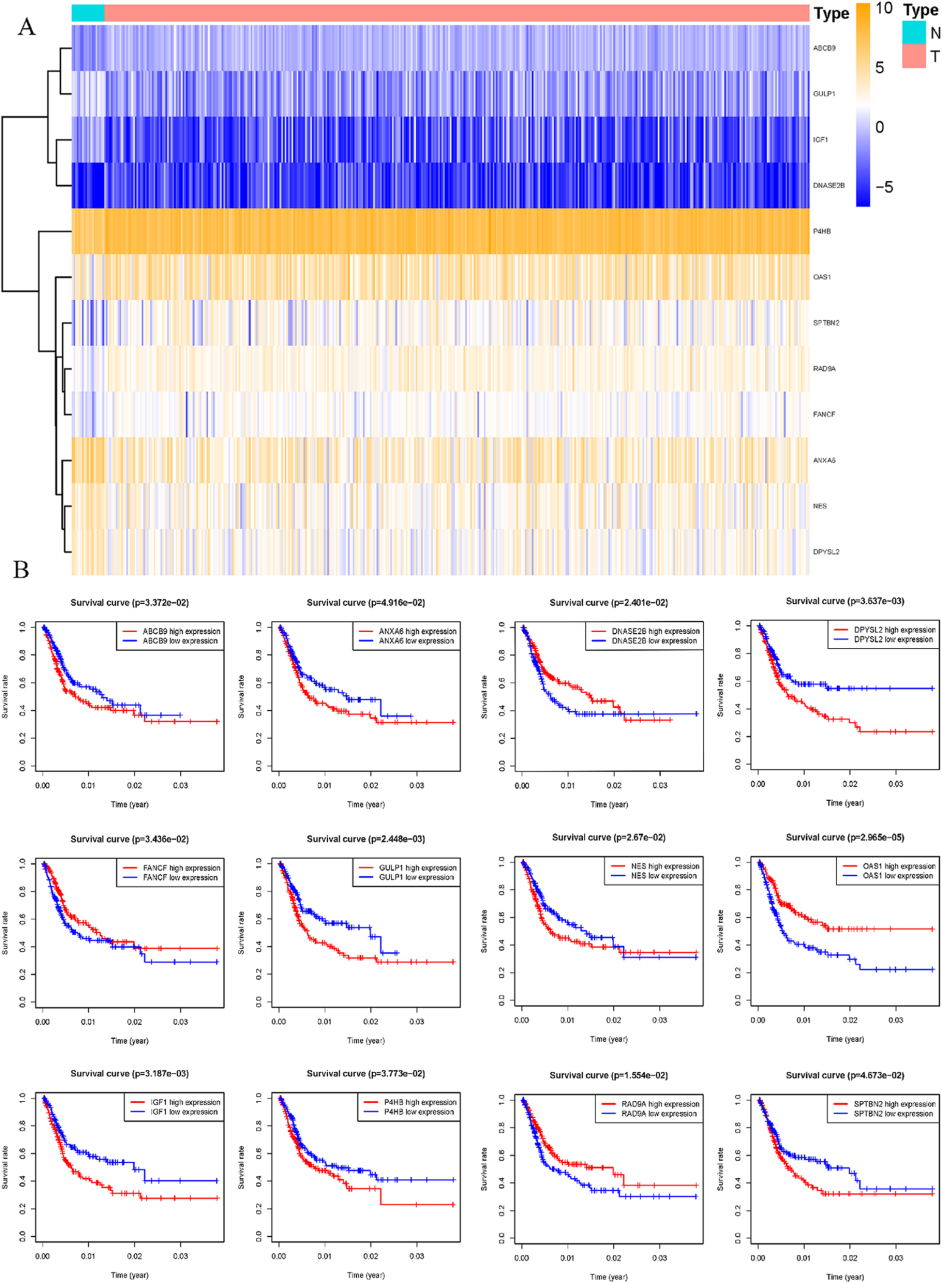
Validation of the prognostic model. **A** Heatmap of multivariate Cox regression analysis of prognostic ARGs. **B** K-M curve of the relationship between OS in BLCA patients and expression levels of 12 screened ARGs

Multivariate COX analysis of 44 apoptosis-related genes found that 12 apoptosis-related genes were significantly associated with the prognosis of bladder cancer patients, namel *ABCB9, SPTBN2, GULP1, OAS1, ANXA6, IGF1, P4HB, RAD9A, DNASE2B, NES, FANCF and DPYSL2*. Re-constructed heat map of these 12 genes (Fig. 4A).By constructing a K-M curve for the selected apoptosis-related genes, we found that the high expression of *ABCB9, SPTBN2, GULP1, IGF1, P4HB, NES and DPYSL2* and the low expression of *ANXA6, OAS1, RAD9a, DNASE2B and FANCF* would be beneficial to the prognosis of bladder cancer patients (Fig. 4B). The risk score of the prognostic model was calculated according to the expression of 12 apoptosis-related genes and the regression coefficient. Risk score = (0.431008782 × ABCB9 expression) + (0.032572276 × SPTBN2 expression) + (0.202248866 × GULP1 expression)- (0.008011578 × OAS1 expression)- (0.010181935 × ANXA6 expression) + (0.289468016 × IGF1 expression) + (0.003311434 × P4HB expression)- (0.051207946 × RAD9a expression)- (1.353718106 × DNASE2B expression) + (0.019875979 × NES expression 27972 × FANCF expression) + (0.023045761 × DPYSL2 expression).

### Validation of the risk prognostic model

**Fig. 5:**
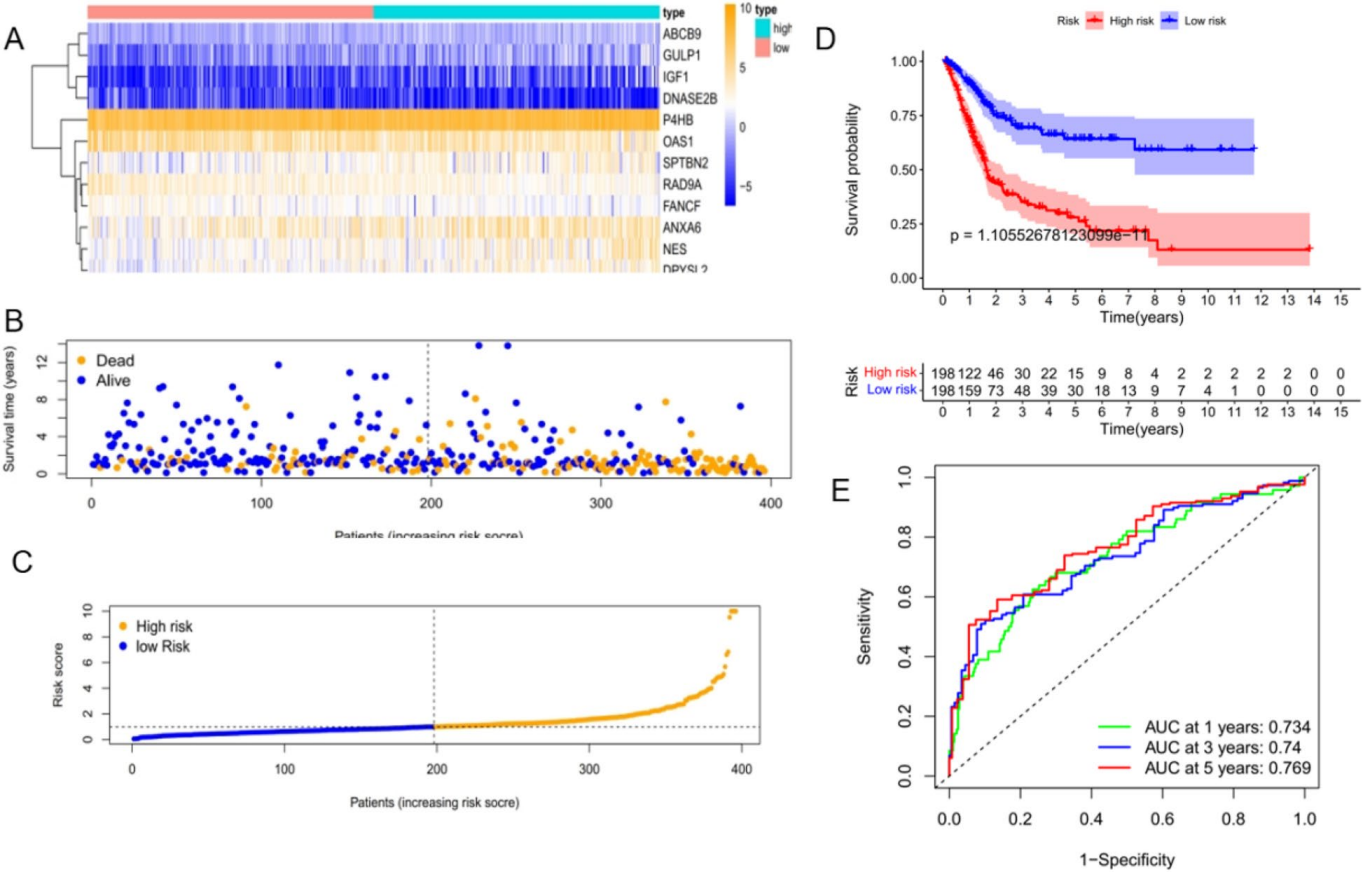
The validation of the reliability of the12-gene signature by analyzing the risk score. **A** Expression heatmap of the twelve identified ARGs in the high and low-risk groups. **B** Risk score distribution in BLCA patients. **C** Scatter plot of survival time. **D** K-M survival plots between high and low-risk groups. **E** ROC curve verified the prognostic performance of the risk score at 1, 3and5 years

**Fig. 6:**
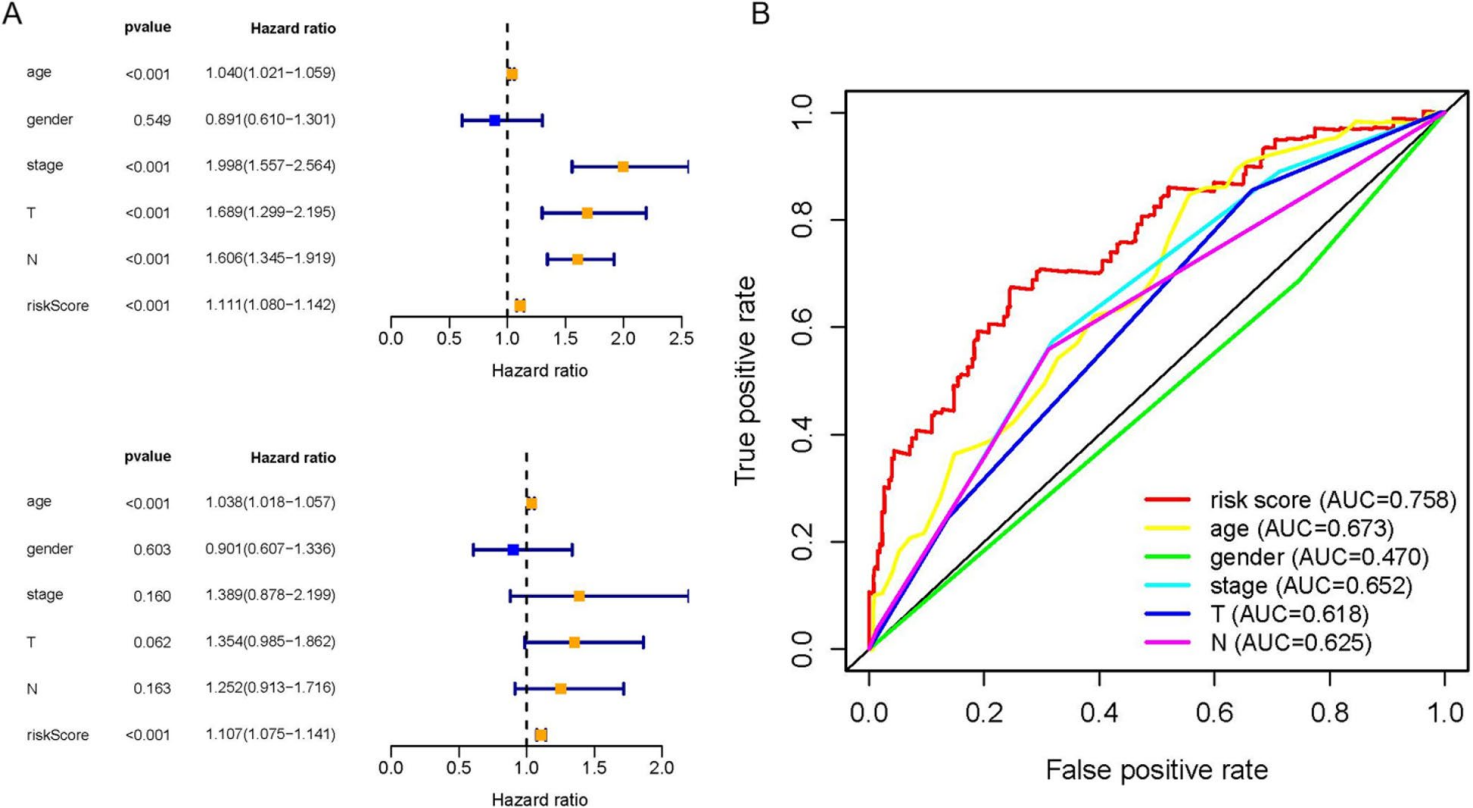
Results of the univariate and multivariate Cox regression analysis regarding OS and ROC curves. **A** Hazard ratios of various clinical variables as determined by univariate and multivariate Cox regression analyses. **B** ROC curves of different clinical factors

**Fig. 7:**
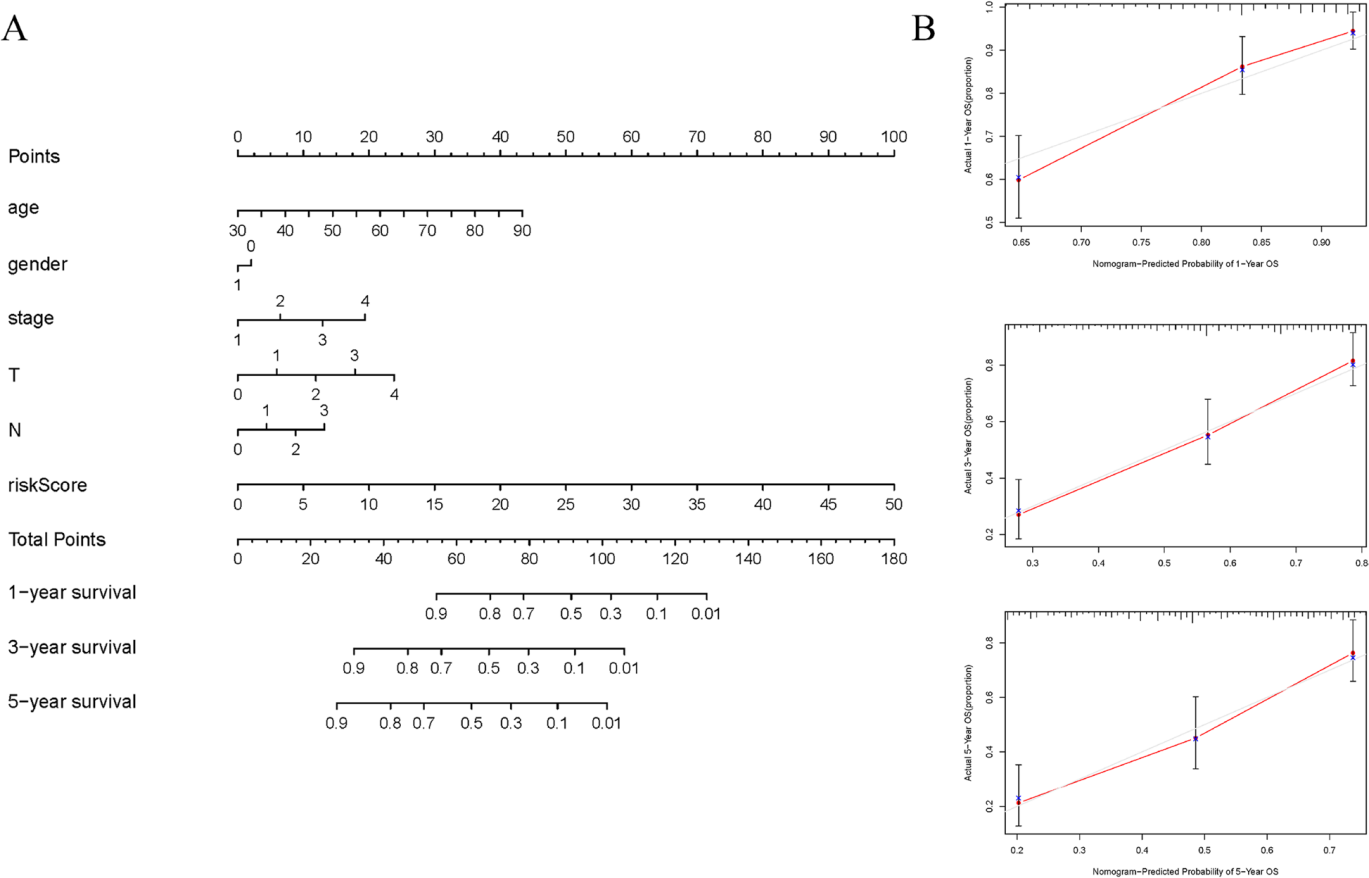
The nomogram constructed based on the prognosis model and verified. **A** The nomogram for predicting the OS of patients with BLCA at 1, 3, and 5 years. **B** Calibration curve of Nomogram diagram at 1, 3and5 years

The patients with bladder cancer were divided into high-risk and low-risk groups according to the risk score calculated from the prognostic model, using the median risk score as a cutoff, and in ascending order (Fig. 5A). The high-risk group had a higher proportion of deaths and a shorter survival time than the low-risk group, indicating a poorer prognosis for the high-risk score group (Fig. 5B). *ABCB9, SPTBN2, GULP1, IGF1, P4HB, NES and DPYSL2* were highly expressed in the high-risk group, suggesting that high expression was positively correlated with high risk; *ANXA6, OAS1, RAD9A, DNASE2B, and FANCF* were under-expressed in the high-risk score group, suggesting a positive correlation between under-expression and high risk (Fig. 5C), consistent with the results of the survival analysis in Fig. 4. Kaplan–Meier survival curves for the high and low risk groups showed a significant difference in OS between the high risk group and the low risk group *(P* < *0.01*, Fig. 5D). The ROC curve (Fig. 5E) showed that the AUC was 0.734 at 1 year, 0.74 at 3 years, and 0.769 at 5 years, all of which had high predictive values. The results showed that the risk score model had a good specificity for predicting the prognosis of bladder cancer. In addition, univariate and multivariate COX analyses of other clinical variables (Fig. 6A) revealed that the risk score had a higher predictive value relative to other clinical variables (Fig. 6B). Finally, we combined the risk scores with the corresponding clinical variables to construct nomograms to predict patients' OS at 1, 3, and 5 years (Fig. 7A), while the calibration curve showed that the nomograms had good predictive power (Fig. 7B).

### External validation of the model

**Fig. 8:**
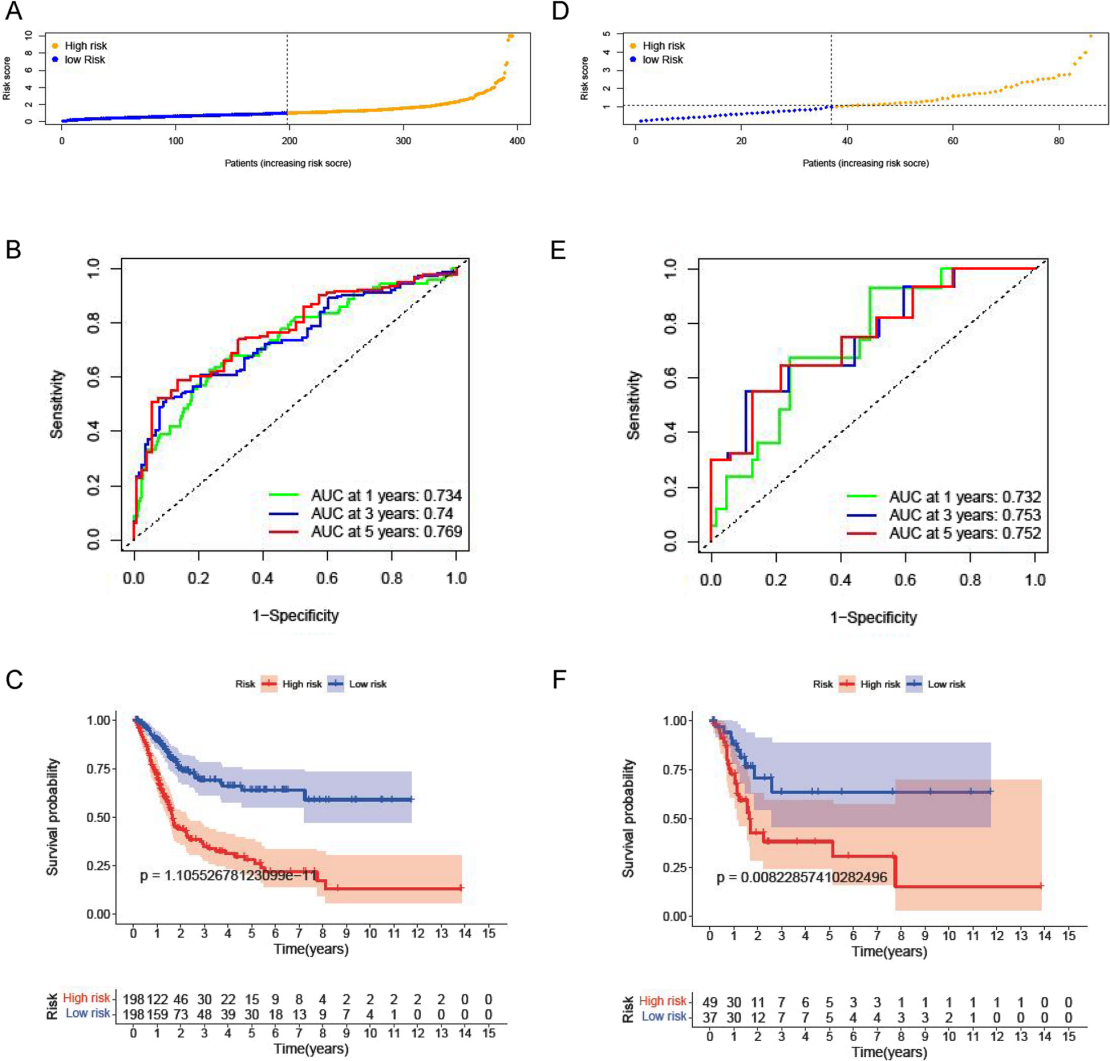
External validation of the model. **A** and **D** Risk score distribution in BLCA patients. **B** and **E** ROC curve verified the prognostic performance of the risk score at 1, 3 and 5 years. **C** and **F** K-M survival plots between high and low-risk groups

External validation analysis using 164 cases of sample data from the BLCA validation set of the GEO database analysis (Fig. 8A), the results showed that the 1,3 and 5 years AUC of the model were 0.734,0.74 and 0.769, suggesting that the model had good predictive ability in the validation set and good extrapolation extrapolation (Fig. 8B). Survival analysis showed that the overall survival of the high-risk group survival analysis was still significantly lower than that of the low-risk group (*P* < *0.01*, Fig. 8C).Similarly, data collected from 86 clinical samples were analysed (Fig. 8D) and the results showed that the 1,3 and 5 years AUC of the model were 0.732,0.753 and 0.752 (Fig. 8E), and the overall survival rate in the high-risk group was significantly lower than that in the low-risk group(*P* < *0.01*, Fig. 8F).

### Immune infiltration level analysis

**Fig. 9:**
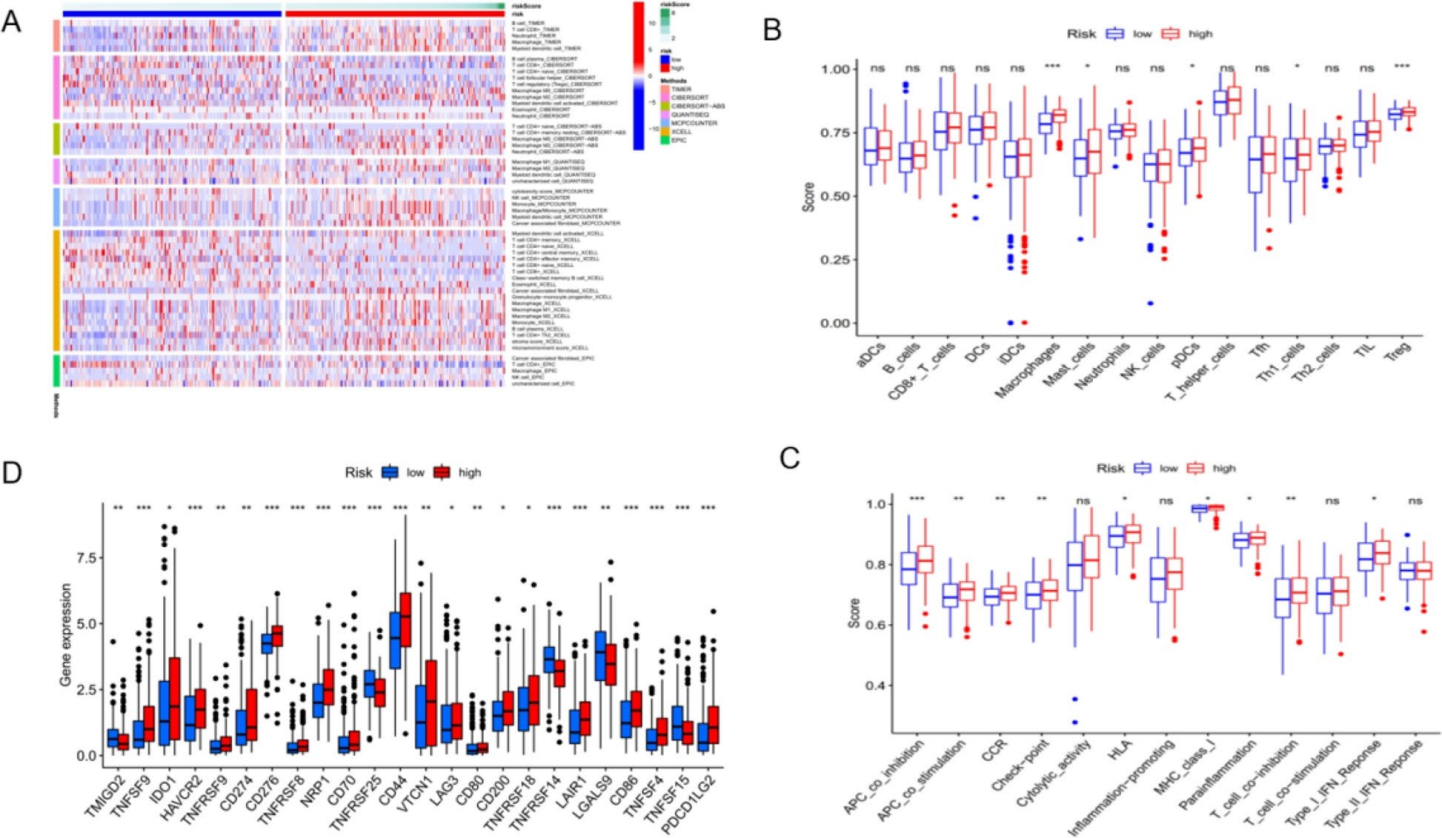
Immunoinfiltration analysis. **A** Heatmap of the correlation of high and low-risk groups with immune characteristics. **B** and **C** Boxplot of relative expression of immune cells and immune function between high and low-risk groups. **D** Expression of immune checkpoints with high and low-risk groups

Based on the Timer Database et al., the heat map of the high and low risk groups associated with immune cells in this prognostic model was drawn (Fig. 9A). The relative abundance of tumor-infiltrating immune cells and immune function in bladder cancer patients quantified by ssGSEA (single sample gene set enrichment analysis) algorithm showed Macrophages, Mast cells, pDCs, Th1 cells,Treg,APC_co_inhibition, APC_co_stimulation, CCR, Check − point, HLA, MHC_class_I, Parainflammation, T _ cell _ Co − inhibition and Type _ I _ IFN _ Reponse were significantly different between the two risk groups *(P* < *0.05*, Fig. 9B and C), and the high-risk group showed higher immunoreactivity. Interestingly, in the correlation analysis of immune checkpoints, we found that almost all immune checkpoints were differentially expressed between the high-risk group and the low-risk group (Fig. 9D), and almost all of the high-risk group showed higher immune suppression. The above results indicate that the model may be related to the immune activity in the tumor immune microenvironment and can show high sensitivity in immunotherapy.

### *P4HB* promote s BLCA progression

**Fig. 10:**
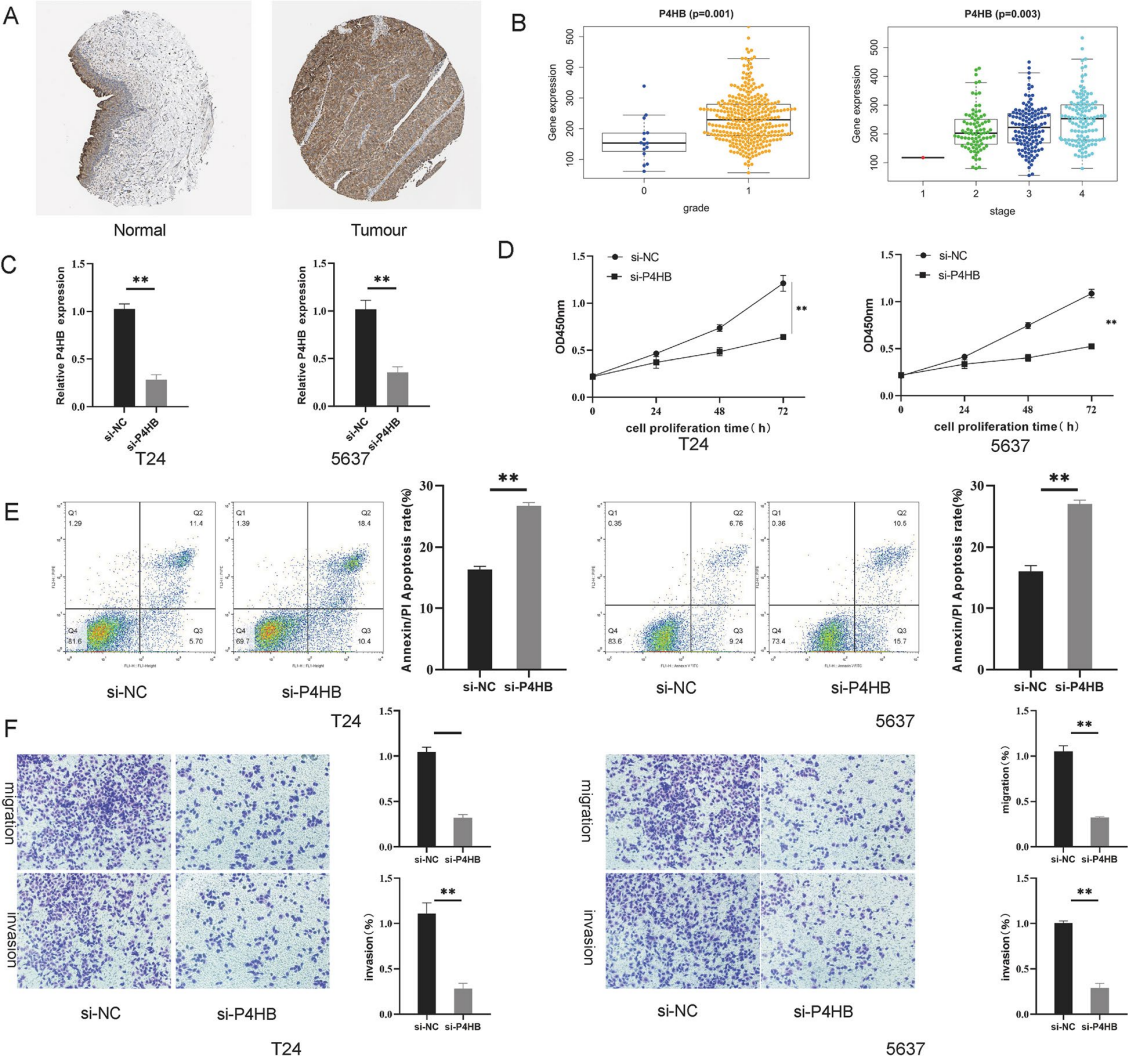
Knockdown of *P4HB* inhibits BLCA progression. **A** Expression of *P4HB* in BLCA tissues and normal tissues. **B** the association between *P4HB* expression and the clinical grade and stage of BC patients. **C** *P4HB* expression in bladder cancer cell s determined by RT-qPCR after knock-down. **D** cell proliferation ability in bladder cancer cells by CCK8. **E** cell apoptosis in bladder cancer cells was detected by flow cytometric analysis (Annexin Fitc/PI). **F** Number of migratory and invasion bladder cancer cells was detected by Transwell assays

In order to further explore the role of these apoptosis-related genes in regulating BLCA cell function, we selected *P4HB* as the subject of our study and performed l-cell function experiments. Initially, we analyzed the protein levels of *P4HB* in clinical BLCA specimens and normal specimens using the Human Protein Atlas (HPA) database (www.proteinatlas.org), and the results were in agreement with the TCGA analysis (Fig. 10A). Meanwhile, the results of the Kruskal test showed that P4HB expression was strongly correlated with the patient grade and stage (Fig. 10B). The si-*P4HB* and the corresponding negative control were transfected into bladder cancer cells and RT-qPCR confirmed that *P4HB* was successfully knocked down (Fig. 10C). CCK8 proliferation assay showed that compared with the si-NC group, the bladder cancer cells in the si-*P4HB* group showed significantly reduced proliferation ability (Fig. 10D); flow cytometry analysis showed increased apoptosis in bladder cancer cells in the si-*P4HB* group (Fig. 10E); in addition, Transwell migration and invasion assays showed that both the migration and invasion ability of the cells was reduced (Fig. 10F).

## Discussion

Bladder cancer is one of the most common malignant tumors of the genitourinary system, most of which originate from the urothelium. Its high recurrence rate and poor prognosis are the difficulties in clinical research. Therefore, it is of great value to develop new methods to evaluate the prognosis of bladder cancer patients. The dysregulation of apoptosis can lead to the initiation and progression of bladder cancer and has predictive potential in the prognosis of some tumors [12]. The rapid development of high-throughput sequencing technology and bioinformatics provides an opportunity to explore new genes involved in the development and progression of bladder cancer and to develop new prognostic models.

Therefore, based on the data of bladder cancer patients in TCGA database, this research screened the differentially expressed apoptosis-related genes in BLCA. Meanwhile, the potential biological processes and pathways of these differentially expressed genes were revealed by GO and KEGG enrichment analysis. Twelve apoptosis-related genes with prognostic significance were obtained by univariate and multivariate COX analysis, and the corresponding risk prognostic model was constructed. The prognostic value of 12 apoptosis-related genes was detected by K-M curve. The results are basically consistent with the analysis in the prognosis model. The high expression of *ABCB9, SPTBN2, GULP1, IGF1, P4HB, NES and DPYSL2* and the low expression of *ANXA6, OAS1, RAD9a, DNASE2B and FANCF* would be beneficial to the prognosis of bladder cancer patients. The ROC curve also shows that the model has a higher predictive value. Combined with other clinical data of patients with bladder cancer, the risk score of this model has the highest predictive power. We also constructed a nomogram to help more intuitively predict the OS of BC patients at 1,3,5 years, and the calibration curve showed its validity.

Among the 12 apoptosis-related genes in the prognostic model, some genes have been shown to play an important role in the occurrence and progression of tumors. *ABCB9* is a member of the ATP-binding cassette subfamily B. The ATP-binding cassette family, as membrane drug transporters, participates in the development of drug resistance in a variety of tumors by allowing anticancer drugs to flow out of cancer cells [13]. At the same time, as an important apoptotic protein, it is down-regulated by miR-31 in cisplatin-resistant non-small cell lung cancer, thus playing an anti-apoptotic role [14]. *ANXA6* (Annexin A6) belongs to the family of calcium-dependent membrane and phospholipid-binding proteins. Like other annexins, *ANXA6* binds to phospholipids and acts in a calcium ion dependent manner, thereby activating the cell membrane in a dynamic, reversible, and regulated manner [15]. *ANXA6* has been identified as a newly synthesized protein in starvation-induced autophagy, and is a novel autophagy regulator that regulates autophagosome formation, which can regulate autophagic by inhibiting the PI3K/AKT/mTOR pathway, thereby improving the therapeutic resistance of nasopharyngeal carcinoma to radiotherapy [16]. *DYPSL2* (dihydropyrimidinase-related protein 2), also known as collapsin response mediator protein 2 (*CRMP2*), belongs to the CRMP family and is capable of promoting tumor progression in multiple cancer types [17]. ZOU et al. found that in bladder cancer, *DYPSL2* can promote aerobic glycolysis and EMT to enhance the growth and metastasis of bladder cancer, which is achieved by combining with *PKM2* [18]. *PKM2* (pyruvate kinase M2) is able to catalyze the final rate-limiting reaction of glycolysis, switching between highly active tetramers and less active dimers in normal cells, but in cancer cells, it often exists in the form of dimer [19]. The binding of *DPYSL2* to *PKM2* can inhibit the formation of tetrameric *PKM2*, resulting in an increase in the level of dimeric *PKM2*, switching glucose metabolism in cancer cells from the normal respiratory pathway to aerobic glycolysis to promote cancer cell proliferation and growth, which is the same as the results in our prognostic model. *FANCF* (Fanconi anemia-related gene F) is a member of the FA protein family, located on chromosome 11p15. This region is rich in cancer-related genes, which is characterized by rich CpG islands and highly methylated [20]. FA protein is a multifunctional protein composed of 15 FA complementary groups, and is involved in cell cycle, DNA damage and repair, apoptosis, gene transcription and gene stability through the FA/BRCA cellular pathway. *FANCF* regulates the FA/BRCA pathway by maintaining the stability of FANC and activating *FANCD2* ubiquitination [21]. Studies have shown that down-regulation of *FANCF* expression can inhibit the proliferation, migration and invasion of breast cancer cells, which may be related to hypermethylation of its promoter [22]. *P4HB* (prolyl 4-hydroxylase B), also known as protein disulfide isomerase, is a multifunctional protein that catalyzes the formation, cleavage and rearrangement of disulfide bonds [23]. In addition, *P4HB* acts as a molecular chaperone and binds to misfolded proteins to prevent their excessive aggregation. As a potential therapeutic target, *P4HB* was found to be a novel diagnostic and prognostic marker for various cancer types, including colon cancer, gastric cancer, and clear cell renal cell carcinoma [24–26]. Previous studies have reported that high *P4HB* expression is significantly related to the poor prognosis of BC patients,inhibition of *P4HB* improve the sensibility of BC cells to Gemcitabine by activating apoptosis and the PERK/eIF2α/ATF4/CHOP pathways [27]. The present study also further confirmed that *P4HB* promotes bladder cancer cell growth.

Many cytokines,antigens,receptors,and cellular components of the immune system are associated with the induction of apoptosis [28, 29], so we explored the relationship between this risk prognostic model and immune-related cells and functions. SSGSEA showed that there were significant differences in the infiltration scores of immune cells and immune functions among different risk groups, and the expression of immune cells and immune functions was significantly increased in the high-risk group. Immune checkpoint inhibitor therapy has become a new promising treatment for BC, so we also analyzed the immune checkpoint in this risk prognosis model. Similar to the results of the infiltration score of immune cells and immune function, the high-risk group has higher expression of immune checkpoint molecules. This suggests that patients in the high-risk group may benefit more from immune checkpoint inhibitor therapy than those in the low-risk group. Taken together, these findings suggest that the poor prognosis of high-risk patients may be related to higher immunoreactivity and immunosuppression, and that apoptosis may play a role in the immunotherapy of BC.

Although this study has successfully constructed a prognostic risk model of apoptosis-related genes for bladder cancer patients, it still has some limitations. First of all, the patient samples in the prognostic model are only from the TCGA database, most of which are European and American, and have not been validated externally. Secondly, other important clinical factors, such as whether there is adjuvant therapy after surgery, may reduce the statistical validity and credibility of the prognostic model. Thus, further statistics are still needed to validate the model.

To sum up, this research determined the expression level of apoptotic genes in bladder cancer and their prognostic impact on patients with bladder cancer through bioinformatics analysis of TCGA bladder cancer database.We found twelve apoptosis-related genes was significantly related to the prognosis of bladder cancer, and constructed a prognostic evaluation model of bladder cancer related to apoptotic genes, which can independently predict the prognosis of patients with bladder cancer. This is worthy of further validation in clinical bladder cancer patients to help clinicians choose precise bladder cancer treatment strategies and evaluate prognostic risks. In addition, this research provides a potential way to predict the efficacy of immunotherapy for bladder cancer in the future.

### Supplementary Information

**Additional file 1: Supplementary Table1.** Human apoptosis-related genes  downloaded from GSEA.

## Data Availability

The data set proposed in this study can be found in the online knowledge base. The name and data set of the repository can be found in the article and supplementary materials.
